# Genomic and Secretomic Analyses Reveal Unique Features of the Lignocellulolytic Enzyme System of *Penicillium decumbens*

**DOI:** 10.1371/journal.pone.0055185

**Published:** 2013-02-01

**Authors:** Guodong Liu, Lei Zhang, Xiaomin Wei, Gen Zou, Yuqi Qin, Liang Ma, Jie Li, Huajun Zheng, Shengyue Wang, Chengshu Wang, Luying Xun, Guo-Ping Zhao, Zhihua Zhou, Yinbo Qu

**Affiliations:** 1 State Key Laboratory of Microbial Technology, Shandong University, Jinan, Shandong, China; 2 Key Laboratory of Synthetic Biology, Institute of Plant Physiology and Ecology, Shanghai Institutes for Biological Sciences, Chinese Academy of Sciences, Shanghai, China; 3 National Glycoengineering Research Center, Shandong University, Jinan, Shandong, China; 4 Shanghai-MOST Key Laboratory of Disease and Health Genomics, Chinese National Human Genome Center at Shanghai, Shanghai, China; 5 School of Molecular Biosciences, Washington State University, Pullman, Washington, United States of America; University of Nottingham, United Kingdom

## Abstract

Many *Penicillium* species could produce extracellular enzyme systems with good lignocellulose hydrolysis performance. However, these species and their enzyme systems are still poorly understood and explored due to the lacking of genetic information. Here, we present the genomic and secretomic analyses of *Penicillium decumbens* that has been used in industrial production of lignocellulolytic enzymes in China for more than fifteen years. Comparative genomics analysis with the phylogenetically most similar species *Penicillium chrysogenum* revealed that *P. decumbens* has evolved with more genes involved in plant cell wall degradation, but fewer genes in cellular metabolism and regulation. Compared with the widely used cellulase producer *Trichoderma reesei*, *P. decumbens* has a lignocellulolytic enzyme system with more diverse components, particularly for cellulose binding domain-containing proteins and hemicellulases. Further, proteomic analysis of secretomes revealed that *P. decumbens* produced significantly more lignocellulolytic enzymes in the medium with cellulose-wheat bran as the carbon source than with glucose. The results expand our knowledge on the genetic information of lignocellulolytic enzyme systems in *Penicillium* species, and will facilitate rational strain improvement for the production of highly efficient enzyme systems used in lignocellulose utilization from *Penicillium* species.

## Introduction

Biorefinery of lignocellulosic biomass to liquid fuels and chemicals is considered to be an important alternative for sustainable development of the human economy and society [1], [2]. Currently, the high cost of lignocellulolytic enzymes (usually referred as lignocellulolytic enzyme systems [3]) is still a major barrier in the production of biofuels from lignocellulosic materials [4]. The fungus *Trichoderma reesei* is now the major source of commercial lignocellulolytic enzyme systems [5]. Although exhibiting high hydrolytic activity on pure cellulose, the enzyme system of *T. reesei* need to be supplemented with several kinds of exogenous enzymes to achieve effective degradation of natural complex lignocellulosic materials [6], [7]. Many *Penicillium* species have been reported to produce native enzyme systems with better performance than that of *T. reesei* (reviewed in [8]). However, systematic studies on these enzyme systems, and work on directed strain engineering are greatly limited, mainly due to the poor knowledge of their genetic backgrounds.

*Penicillium decumbens* has been used for industrial-scale cellulase production in China since 1996 [9]. The wild strain was isolated from decayed straw-covered soil in 1979 [10]. After a long-term strain improvement process [10], [11], a high cellulase productivity of 160 filter paper units L^−1 ^h^−1^ was reached by *P. decumbens* mutant strains. Because of its more balanced enzyme composition (e.g. markedly higher β-glucosidase activity [12]) compared with that of *T. reesei*, the enzyme system of *P. decumbens* has been used in laboratory- or pilot-scale production of ethanol and hydrogen from lignocellulosic materials [13]–[15]. Furthermore, the enzyme system of *P. decumbens* shows good performances in pulping for its high xylanase activity [16], and in extraction of plant flavonoids for its high transglycosylation activity [17].

More than ten cellulase components have been purified [18]–[20], and several cellulase-encoding genes have been cloned from *P. decumbens* [21], [22]. Some of the enzymes have unique characters, and show synergy with the enzyme system from *T. reesei* to decompose natural lignocellulosic biomass [19], [23]. However, a comprehensive understanding of the lignocellulolytic enzyme system of this fungus is still lacked. Here, we report the genome sequence of *P. decumbens* wild-type strain 114-2, which represents the first genome of a high lignocellulolytic enzymes-producing species in the genus *Penicillium*. Comparative genomic analysis with *T. reesei* suggested a more diverse set of lignocellulolytic enzyme-encoding genes in *P. decumbens*. In addition, the secretomes of *P. decumbens* were analyzed to identify specifically expressed proteins under different culture conditions.

## Results and Discussion

### The *P. decumbens* Genome Consists of Eight Putative Chromosomes and a Circular Mitochondrial DNA

Based on combined data from 454 GS FLX Titanium sequencing (∼28-fold coverage) and ABI SOLiD mate-paired library sequencing, the 30.19-Mb genome of *P. decumbens* 114-2 was assembled into nine scaffolds comprising 344 contigs (**Table S1**). Telomeric repeats (primarily 5′-TTAGGGG-3′) were found at the ends of eight large scaffolds, resembling the eight chromosomes (**Table S2**). The remaining smaller circular scaffold of 26.36 kb was more likely a mitochondrial genome. The number of putative chromosomes is the same as those in some sequenced *Aspergillus* species, such as *A. nidulans* [24] and *A. niger* [25], but different from that in *P. chrysogenum* (four, determined by pulsed-field gel electrophoresis [26]).

Gene curation from four gene predictors yielded 10,021 protein-coding gene models (**Table S3**), of which 35.2% were well supported by 454 transcriptome sequencing data (100% identity in full-length; **Table S4**). Totally, 6,186 proteins were assigned to Gene Ontology (GO) categories [27]. The genome size and number of protein-encoding genes of *P. decumbens* 114-2 were comparable to those of other sequenced filamentous fungi (**Table S5**). The general features of the *P. decumbens* genome were summarized in **Table 1**.

**Table 1:** General features of the *P. decumbens* 114-2 genome.

Genomic features	Value
**Nuclear genome**	
Size of assembled genome (Mbp)	30.2
GC content of assembled genome (%)	50.6
All protein-coding genes	10,013
Protein-coding genes (≥60 aa)	9,823
GC content of protein-coding region (%)	54.4
Average gene length (bp)	1,597
Average number of introns per gene	1.95
Genes with intron	7,766
Average intron size (bp)	118
Average exon size (bp)	463
Number of tRNA genes	176
**Mitochondrial genome**	
Size (bp)	26,362
GC content (%)	26.4
Protein-coding genes	8
Number of tRNA genes	29

### The Cellular Metabolism and Regulatory Network of *P. decumbens* is Less Complex than its Phylogenetically most Related *P. chrysogenum*

**Figure 1:**
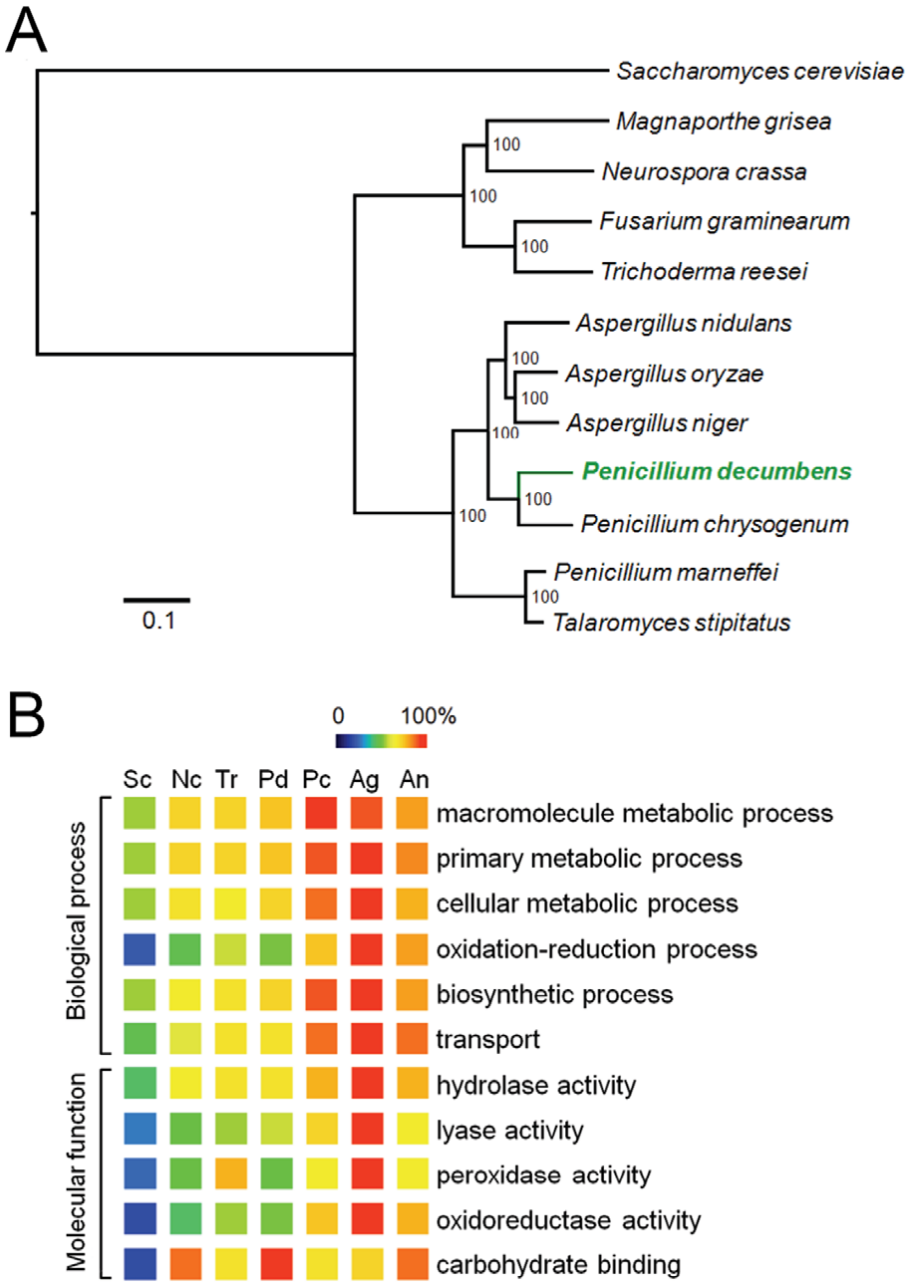
Comparative genomic analysis of *P. decumbens* and other fungal species. (**A**) Maximum-likelihood phylogenetic tree of *P. decumbens* and eleven Ascomycota species. (**B**) Comparison of number of proteins in selected Gene Ontology terms (level 3) involved in carbohydrate utilization and cellular metabolism. The maximum number in each term was set to be 100%. Sc, *Saccharomyces cerevisiae*; Nc, *Neurospora crassa*; Tr, *Trichoderma reesei*; Pd, *P. decumbens*; Pc, *P. chrysogenum*; Ag, *A. niger*; An, *A. nidulans*.

The genome sequences of three species have been published in the genus *Penicillium*, including those of penicillin-producing *P. chrysogenum* [28], the conditional human pathogen *P. marneffei* and the rotting wood isolate *P. stipitatum* (*Talaromyces stipitatus*). A phylogenetic tree based on 1,760 single-copy orthologs (**Table S6**) established that *P. decumbens* was more closely related to *P. chrysogenum* than to *P. marneffei* and *P. stipitatum* (**Figure 1A**). The relative close relationship between *P. decumbens* and *P. chrysogenum* was also supported by analyzing the top hits of BLASTp search of *P. decumbens* proteins in the NCBI non-redundant protein database (**Figure S1A**). *P. decumbens* and *P. chrysogenum* shared 7,035 orthologous proteins with an average amino acid sequence identity of 69.3%. The protein identity was similar to that among *A. nidulans*, *Aspergillus fumigatus* and *Aspergillus oryzae* (66–70% [24]), and lower than that among *T. reesei*, *Trichoderma atroviride* and *Trichoderma virens* (70–78% [29]).

**Figure 2:**
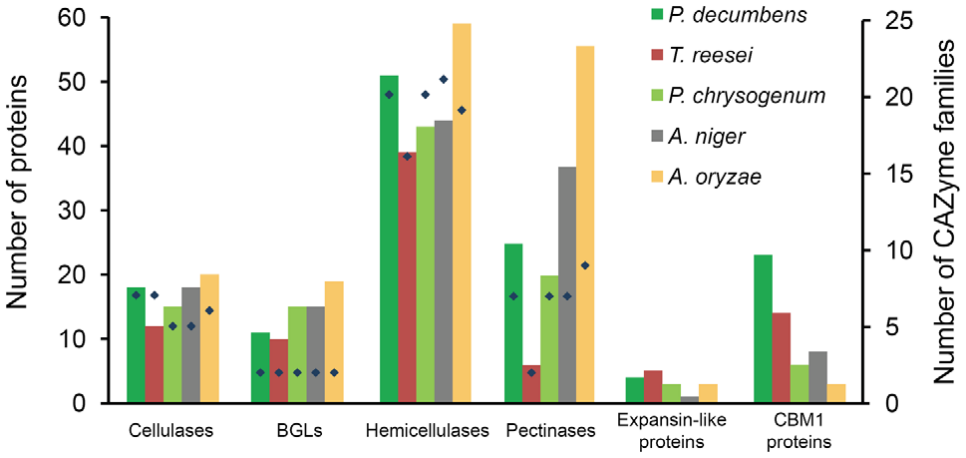
Comparison of numbers of plant cell wall-degrading enzymes among five fungal species. BGLs, β-glucosidases. CBM1 proteins, proteins containing fungal cellulose binding domains. Numbers of proteins (columns) and corresponding CAZyme families (diamonds, only those of cellulases, β-glucosidases, hemicellulases and pectinases) are shown. Tannases, cellobiose dehydrogenases and feruloyl esterases not assigned to CAZy families (see **Table S10**) are not included.

Notably, the predicted proteome of *P. decumbens* was 21.7% smaller than that of *P. chrysogenum*. Homologous gene family analysis suggested that the difference was mainly due to the higher number of species-specific genes in *P. chrysogenum*, and to a less extent, due to the expansion (in *P. chrysogenum*) or contraction (in *P. decumbens*) of shared gene families between the two species (**Figure S1B**). Comparison of protein Gene Ontology (GO) classifications indicated that *P. decumbens* had clearly fewer proteins involved in cellular metabolism, biosynthesis and transport than *P. chrysogenum* (**Figure 1B** and **Table S7**). The result was also confirmed by comparison of the numbers of some functional proteins, such as short-chain dehydrogenases, cytochrome P450s, secondary metabolism key enzymes and membrane transporters, between the two species (**Table S8**). In addition, *P. decumbens* had 35.4% fewer protein kinases and 18.5% fewer transcription factors than *P. chrysogenum*, respectively (**Table S8**). On the other hand, *P. decumbens* was rich with proteins containing carbohydrate-binding domains (**Figure 1B**) and involved in plant cell wall degradation (see **Figure 2** in next section) compared with *P. chrysogenum*. When compared with five other fungal species, the higher number of carbohydrate binding proteins in *P. decumbens* was also highlighted (**Figure 1B**). Interestingly, the numbers of proteins involved in cellular metabolism and regulation in *P. decumbens* were similar to those in *T. reesei*. We speculate that a relatively simple cellular metabolism network might be more suitable for high-level production of extracellular lignocellulolytic enzymes.

### *P. decumbens* has more Lignocellulolytic Enzymes in Number and Kind than *T. Reesei*

Natural lignocellulosic substrates are complex in structure and composition and thus require multiple enzymes with diverse substrate specificities for efficient hydrolysis [30]. A total of 371 carbohydrate-active enzymes (CAZymes) [31] were annotated in *P. decumbens*, of which 81 enzymes were predicted to be involved in lignocellulose degradation (**Table S9**). The number and predicted substrate specificities of these enzymes were then compared with those in *T. reesei* as well as three related species including *P. chrysogenum*, *A. niger* and *A. oryzae* (**Figure 2**).

**Figure 3:**
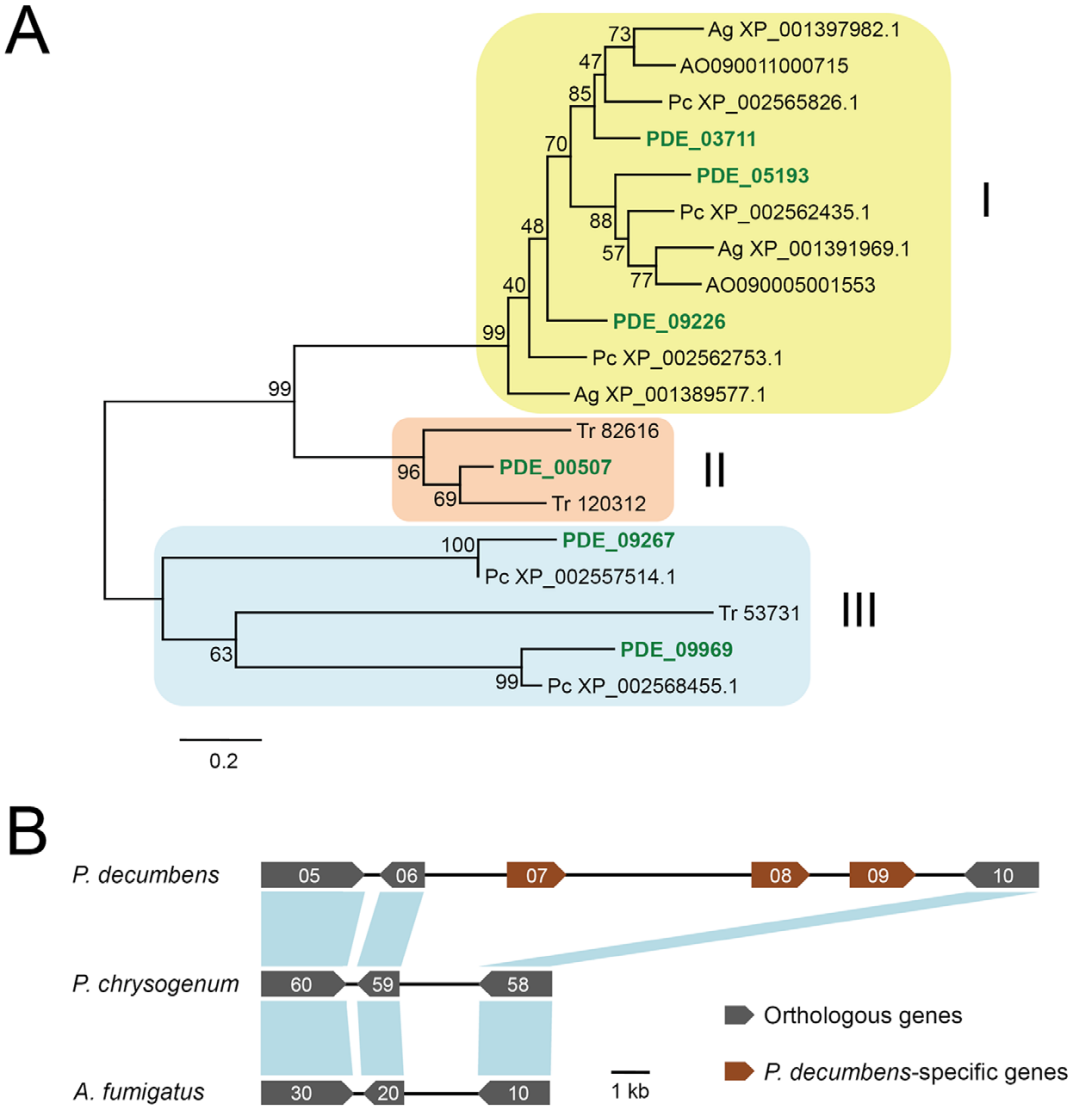
Comparative genomic analysis of GH family 5 EGs among fungal species. (**A**) Maximum likelihood phylogenetic tree of catalytic domains of GH family 5 EGs in *P. decumbens* (in green color), *T. reesei* (Tr), *P. chrysogenum* (Pc), *A. niger* (Ag) and *A. oryzae* (AO). Two EGs in *A. oryzae* (AO090003001342 and AO090005000423) showing sequence identity with cluster III members were not included in the analysis for possible incorrect protein prediction. (**B**) Syntenic analysis of the genomic region around the PDE_00507 gene among *P. decumbens* (scaffold_1∶1,391,035-1,411,127), *P. chrysogenum* (contig Pc00c16∶3,512,265-3,508,864) and *A. fumigatus* (chromosome_1∶4,317,818-4,314,645). Suffixes of gene IDs or GenBank accession numbers (PDE_005** for *P. decumbens*, XP_0025617** for *P. chrysogenum* and Afu1g159** for *A. fumigatus*) are shown in panel B.

Eighteen cellulases, including three cellobiohydrolases (CBHs), eleven endo-β-1,4-glucanases (EGs) and four cellulose monooxygenases (CMOs [32], previously known as glycosyl hydrolase (GH) family 61 EGs) were predicted to be encoded in the *P. decumbens* genome. The numbers were all higher than those in *T. reesei* (**Table S10**). Especially, *P. decumbens* possessed six EGs in GH family 5, whereas *T. reesei* has only three. We then performed a phylogenetic study on GH family 5 EGs from the five fungal species. While *P. decumbens* had members in all three clusters, the other four species had members in only one or two clusters (**Figure 3A**). Notably, one GH family 5 EG, PDE_00507, had no ortholog in all other species with published genome sequences in the family Trichocomaceae (data not shown), and seemed to be acquired by a horizontal gene transfer event (**Figure 3B**). For β-glucosidase which is important in the release of product inhibition of cellulases [12], *P. decumbens* had eleven genes. In addition to the previously characterized BGLI (PDE_02736) [33], three additional secreted β-glucosidases were predicted, including one in GH family 1 and two in GH family 3 (**Table S9**).

Hemicellulose closely associated with cellulose and lignin is another principle component in lignocellulosic biomass [7]. *P. decumbens* was also rich in hemicellulose-degrading enzymes, with 51 related enzymes assigned to 20 CAZyme families predicted, in contrast to 39 to 16 families in *T. reesei*. The hemicellulases in *P. decumbens* were divided into 11 types according to their substrate specificities (**Table S10**). Also, many more pectinases (25 genes) were found in the *P. decumbens* genome than in *T. reesei* (6 genes) (**Table S10**). Significantly, *P. decumbens* possessed genes encoding some enzymes that are important for lignocellulose degradation but absent in *T. reesei* [34]. These included five feruloyl esterases, three tannases, one endo-β-1,4-galactanase, and one cellobiose dehydrogenase (**Table S10**). Thus, the enzyme system of *P. decumbens* may be more efficient in the degradation of natural lignocellulosic materials. For example, feruloyl esterases may facilitate the hydrolysis of cellulose by hydrolyzing the ester bonds cross-linking lignin and xylan, as reported in the saccharification of wheat straw [35]. Xyloglucanases in GH family 74 and glucuronoyl esterases were not found in *P. decumbens*, but there remains the possibility that some proteins in GH families 5 and 12 may exhibit hydrolytic activities on xyloglucan [36].

When compared with the three related species, *P. decumbens* had more cellulases, hemicellulases and pectinases than *P. chrysogenum*, more hemicellulases but fewer pectinases than *A. niger*, and fewer cellulases, hemicellulases and pectinases than *A. oryzae*. Compared with the two recently sequenced thermophilic fungi *Myceliophthora thermophila* and *Thielavia terrestris* [37], *P. decumbens* had fewer CBHs, CMOs and xylanases. The results revealed extensive lineage-specific gain and loss of plant cell wall-degrading genes in fungi. Furthermore, it should be noted that the results of comparison of degrading enzymes did not exactly correspond with those of related CAZyme families and enzyme types (**Figure 2** and **Table S10**). For example, despite the higher total number of proteins, both the cellulases and hemicellulases in *A. oryzae* belonged to fewer CAZyme families than those in *P. decumbens*, respectively. As enzymes from different CAZyme families could play synergistic or complementary roles in the degradation of the same substrate [38], [39], we propose that the completeness and diversity of enzyme systems should be emphasized in future development of highly efficient plant cell wall-degrading enzyme systems.

Totally, 23 genes encoding proteins with cellulose-binding domain (family 1 carbohydrate binding module, CBM1) were found in *P. decumbens*, higher than that (14 genes) in *T. reesei*. In fact, *P. decumbens* had the highest number of CBM1-containing proteins among all sequenced *Aspergillus* and *Penicillium* species (**Fig. S2**). The fact implied that more enzymes of *P. decumbens* could be attached to the surface of insoluble lignocellulosic substrates to achieve a more efficient degradation. Particularly, CBM1s were present in up to twelve hemicellulases in *P. decumbens*, which may facilitate the degradation of hemicelluloses in natural cellulose-hemicellulose networks [40]. On the other hand, it should be noted that some fungi had higher numbers of CBM1s than *P. decumbens*. For example, a cellulase high-producing *M. thermophila* isolate was reported to have up to 46 CBM1s according to the annotation of its genome [41].

Similar to that in *T. reesei* [34], genomic regions encoding higher densities of CAZyme genes were also observed in *P. decumbens*. We further analyzed the genome locations of all 114 genes encoding plant cell wall-degrading enzymes (including cellulases, hemicellulases, β-glucosidases, pectinases, expansin-like proteins [42], tannases and cellobiose dehydrogenase). Notably, eight genomic areas with a total length of 0.72 Mb (2.4% of the genome) encoded 31 plant cell wall-degrading enzymes (27.2% of total; **Figure S3** and **Table S11**). Of the eight areas, seven were located in sub-telomeric regions (within 200 kb from putative chromosome ends), suggesting that they might have been recently translocated onto these regions to adapt to a lignocellulose-rich environment [43]. In addition, adjacent locations of plant cell wall-degrading enzyme genes were frequently observed in the genome of *P. decumbens* (eight pairs and two triplets; **Table S11**). None of such adjacent genes were found to be paralogs, ruling out the possibility of formation of these “gene clusters” through gene duplication.

### The Composition of *P. decumbens* Secretome Varied in Different Culture Media

With the combination of a series of computational tools (see Materials and Methods), the *P. decumbens* genome was predicted to encode 512 secreted proteins, including 84 of the 114 predicted plant cell wall-degrading enzymes. Also, chitinases, proteases, oxidoreductases, and 183 proteins of unknown function were included in the predicted secretome (**Table S12**). To assess the expression levels of these secreted proteins, the secretomes of *P. decumbens* grown in two different media were analyzed using the two-dimensional difference in gel electrophoresis (DIGE) technique. The glucose medium is a repressing condition, while the cellulose-wheat bran (CW) medium is a inducing condition for the expression of cellulases and hemicellulases in *P. decumbens* [44], [45]. After a 48-h cultivation, significantly higher lignocellulolytic enzyme activities and a ∼two-fold higher total protein concentration were observed in the culture supernatant of the CW medium than that of the glucose medium (**Figure S4**).

**Figure 4:**
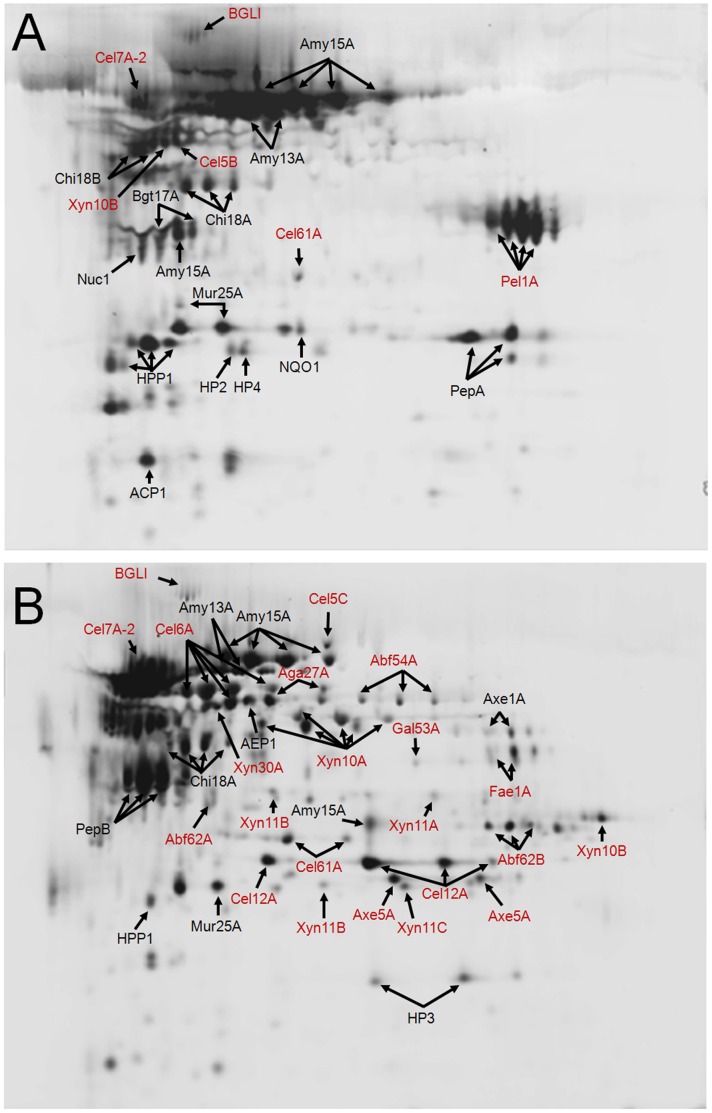
DIGE analysis of *P. decumbens* secretomes. Extracellular proteins of *P. decumbens* grown in glucose medium (**A**) and in cellulose-wheat bran medium (**B**) for 48 h were analyzed at equal protein load. Plant cell wall-degrading enzymes are indicated in red color. Protein IDs and spot abundance changes are listed in **Table S13**.

In total, 101 protein spots on the DIGE gels were successfully identified and assigned to 37 protein models using MALDI-TOF-TOF mass spectrometry **(** **Figure 4** and **Table S13**). When several protein spots were assigned to one protein, they had similar molecular weights but different isoelectric points, suggesting that they were more likely isoforms caused by heterogeneous post-translational modifications. Among the 37 proteins identified, 21 are involved in the degradation of plant cell wall polysaccharides. Other biopolymer-degrading enzymes including amylases, proteases, chitinases and a lysozyme were also identified, suggesting the potential of *P. decumbens* as a versatile cell factory for the production of extracellular enzymes. In addition, the identification of a putative phytotoxic protein (PDE_03255, cerato-platanin family protein [46]) in the secretome hinted possible plant pathogenicity of *P. decumbens*.

The secretome composition of *P. decumbens* differed markedly between the two media. First, cellulases and hemicellulases increased remarkably in the CW medium compared with those in the glucose medium, both in kinds and in contents. Notably, the cellobiohydrolase Cel7A-2 was calculated to account for ∼16% of the total proteins in the CW medium, much higher than that in the glucose medium (∼2.5%). Unlike those of cellulase and hemicellulases, the proportions of BGLI, the only detected β-glucosidase in the secretome, were not much different between the two secretomes (spot abundance changes <2). Second, two amylases, Amy15A and Amy13A, were highly abundant in both secretomes. This is consistent with the results of amylase assays (**Figure S4E**), suggesting a less strict repressing effect of glucose on the two amylases than on cellulases and hemicellulases. Third, fine-tuned expression of proteases in response to different media was observed, with a metalloprotease PepA mainly expressed in the glucose medium and an aspartic protease PepB specially expressed in the CW medium. Finally, a pectate lyase Pel1A was highly detected (∼12.1% of total proteins) in the secretome in the glucose medium but completely absent in the CW medium, suggesting a different regulatory mechanism for this pectin-degrading enzyme from cellulases and hemicellulases. In summary, *P. decumbens* was able to efficiently express some (but not all) lignocellulolytic enzymes in response to lignocellulose in the medium, while expressions of other secreted proteins were regulated in diverse manners.

We further compared the secretome of *P. decumbens* in the CW medium with the reported secretome of *T. reesei* [47]. Although different inducers were used, both the secretomes were composed of primarily cellulases and hemicellulases, with a GH family 7 CBH identified as the most abundant component. On the other hand, more kinds and higher amounts of hemicellulases were contained in the *P. decumbens* secretome than those in *T. reesei*. Particularly, up to six genetically-different endo-β-1,4-xylanases were observed. It was also notable that endo-β-1,4-galactanase and feruloyl esterase, absent in the *T. reesei* genome [34], were detected in the *P. decumbens* secretome. These hemicellulases may act synergistically with cellulases in the hydrolysis of natural lignocellulolytic materials [48]. Comparison of the secretome compositions also indicated some possible directions for genetic engineering of *P. decumbens*. For instance, β-xylosidases not detected in the *P. decumbens* secretome (although five were detected in the transcriptome, **Table S9**) might need to be over-expressed. In addition, considerable amounts of amylases and protease were present in the *P. decumbens* secretome but rarely detected in that of *T. reesei*. Elimination of these irrelevant proteins by gene deletion was expected to relieve the pressure of protein synthesis and secretion and to improve the expression levels of lignocellulolytic enzymes in *P. decumbens*.

### The Cellular Machinery for Lignocellulolytic Enzymes Production in *P. decumbens*

The cellular machineries for lignocellulolytic enzymes production have been reported to be somewhat different among fungal species [49]. We performed an orthology search of known proteins involved in the regulation of lignocellulolytic genes in fungi [5] against the translated proteins in the *P. decumbens* genome. Orthologs of most known regulators were identified in *P. decumbens* (**Table 2** and **Table S14**), including the carbon catabolite repressor CreA/CREI (PDE_03168), cellulase transcription activator CLR-2 (PDE_05999) and protein methyltransferase Lae1 (PDE_00584). However, some related proteins, including ACEII, Xpp1, BglR, ENVOY and GRD1, have no ortholog in *P. decumbens*. We conclude that *P. decumbens* may use both fungal-conserved and unknown mechanisms for the regulation of lignocellulolytic genes.

**Table 2:** Ortholog distribution of characterized transcription factors involved in regulation of lignocellulolytic genes in *P. decumbens* and *T. reesei*.

Protein name	GenBank accession No.		Regulatory role^a^	Reference(s)		Protein ID of orthologs	
						*P. decumbens*	*T. reesei*
**Cellulase and hemicellulase regulators**							
XlnR (*A. niger*)		O42804	+		[87]	PDE_07674	122208
ACEII (*T. reesei*)		Q96WN6	+		[88]	Not found	78445
CLR-1 (*Neurospora crassa*)		EAA33055	+		[89]	PDE_04046	27600
CLR-2 (*N. crassa*)		EAA33476	+		[89]	PDE_05999	26163
ClbR (*Aspergillus aculeatus*)		BAM36047	+		[90]	PDE_04311	Not found
AreA (*A. nidulans*)		P17429	+		[91]	PDE_03334	76817
CREI (*T. reesei*)		CAA64655	–		[92]	PDE_03168	120117
ACEI (*T. reesei*)		AAF35286	–		[93]	PDE_01988	75418
PacC (*A. nidulans*)		Q00202	Gene-dependent		[94]	PDE_05163	120698
HAP2 (*T. reesei*)		AAK69170	Gene-dependent		[95], [96]	PDE_06383	124286
HAP3 (*T. reesei*)		AAK68862	Gene-dependent		[95], [96]	PDE_02832	121080
HAP5 (*T. reesei*)		AAK68863	Gene-dependent		[95], [96]	PDE_01016	AAK68863^b^
Xpp1 (*T. reesei*)		EGR46848	Not reported		[97]	Not found	122879
**β-Glucosidase regulator**							
BglR (*T. reesei*)		EGR44729	+	[98]		NO	52368

a Plus for positive effect and minus for negative effect.

b Missed in genome-wide protein prediction.

The protein secretion pathway in *P. decumbens* was roughly similar to that in *T. reesei* QM6a, with some differences observed in the protein glycosylation pathway (**Table S15**). Although their biological significances have yet to be studied, the number and sequence of some enzymes putatively associated with protein glycosylation, such as α-glucosidases, α-mannosyltransferases, α-mannosidases and α-N-acetylglucosaminyltransferases, varied to different extents between the two species. Therefore, glycans attached to secreted proteins from *P. decumbens* may differ from those in *T. reesei*. Considering that glycosylation may significantly affect the enzymatic properties of lignocellulolytic enzymes (as previously reported for CBHI in *P. decumbens* [18]), the nature and regulation of protein glycosylation in *P. decumbens* are to be investigated and engineered in the future.

### Conclusion

The determination of *P. decumbens* genome has expanded our understanding on the diversity of the composition of lignocellulolytic enzyme systems in fungi. Comparative genomic analysis suggested that *P. decumbens* is a promising species for production of lignocellulolytic enzyme systems for biomass utilization. Based on the information provided by genomic and secretomic analysis, our future work in *P. decumbens* will be centered on strain engineering for higher extracellular protein yield and better hydrolysis performance of the enzyme system. With the aid of the previously developed highly efficient gene targeting system [50], functional genomics studies are being performed to investigate the regulatory mechanisms for lignocellulolytic genes in *P. decumbens*.

## Materials and Methods

### Strains and Culture Conditions

*Penicillium decumbens* 114-2 was stored at Shandong University, and has been deposited at the China General Microbiological Culture Collection Center (CGMCC) under the number of CGMCC 5302. The strain was grown on wheat bran extract slants [51] at 30°C for 4 days for sporulation. In all cultivations, the conidial suspension was inoculated at a final concentration of 10^6^ per ml, and was grown at 30°C with rotatory shaking at 200 rpm. For genomic DNA isolation, the strain was grown in Sabouraud dextrose broth plus yeast extract medium (1% yeast extract, 1% tryptone and 4% dextrose, wt/vol) for 48 h. For 454 transcriptome sequencing, the strain was grown in the following three media respectively for 36 h: modified Mandels’ salt solution (MMS) supplemented with 2% (wt/vol) glucose, MMS with 4% (wt/vol) microcrystalline cellulose, or MMS with 4% (wt/vol) microcrystalline cellulose plus 2% (wt/vol) wheat bran. The composition of MMS was (g L^–1^): KH_2_PO_4_ 3.0, (NH_4_)_2_SO_4_ 2.0, MgSO_4_·7 H_2_O 0.5, CaCl_2_ 0.5, FeSO_4_·7 H_2_O 0.0075, MnSO_4_·H_2_O 0.0025, ZnSO_4_·7 H_2_O 0.0036 and CoCl_2_·6 H_2_O 0.0037. For enzyme assays and secretome analysis, the strain was grown in MMS supplemented with 0.1% (wt/vol) peptone and 1% (wt/vol) glucose, and MMS with 0.1% (wt/vol) peptone, 1% (wt/vol) microcrystalline cellulose and 1% (wt/vol) wheat bran (CW medium), respectively. Samples were taken at different time points for enzyme assays as indicated in **Figure S4**. According to the result of one dimensional SDS-PAGE analysis, the compositions of secretomes from 48 h to 120 h were about the same, respectively, for both cultures. Thus, cultures at 48 h were used for secretomic analysis.

### Genome Sequencing, Assembly and Scaffolding

The genome was sequenced with Roche 454 GS FLX Titanium platform and ABI SOLiD. 35 bp-long reads were produced by ABI SOLiD mate-paired sequencing of DNA libraries with inserts from 3 kb to 5 kb. The standard 454 assembler Newbler [52] version 2.3 was used to generate contigs. ABI SOLiD reads were mapped to contigs from 454 assembler with SHRiMP [53] version 1.3.0 to generate scaffolds with customized pipeline implemented with scaffold analyzer ConPath [54] algorithm. Raw 454 reads were also assembled using several other 454 assembler including WGS 6.1 [55], MIRA 3.2.1 [56], Phrap (http://www.phrap.org/phredphrap/phrap.html) and Newbler 2.0. The generated contigs were used to fill the gaps in the scaffold with Phrap and Minimus2 [57].

### Transcriptome Sequencing and Assembly

cDNAs were synthesized from total RNA samples and then sequenced by 454 GS FLX Titanium platform. The raw 454 reads were assembled using 454 Newbler assembler 2.3 with cDNA parameter.

### Gene Finding and Annotation

Gene models were predicted independently with a set of gene finders including Augustus [58], GeneMark-ES [59], GeneId [60] and SoftBerry eukaryotic gene finding suite (Fgenesh and Fgenesh+) [61]. Augustus parameters were trained on gene models in *Aspergillus* (*A. fumigatus*, *A. nidulans*, *A. oryzae* and *A. terreus*) and *Penicillium* (*P. chrysogenum*) with the transcriptome data as hints. GeneMark-ES works as a self-training manner. The available fungal parameter *A. nidulans* was used for GeneId gene predictor. Gene models generated from the gene predictors were manually curated with 454 transcriptome information and nr BLAST [62] hits to justify intron-exon boundaries and UTR regions.

BLASTp against NR database, UniProt/SwissProt [63], KEGG 57 [64] and String 8.2 [65] were performed to assign general protein function profiles. PFAM 24.0 [66], SUPERFAMILY 1.73 [67], SMART 6 [68] and TIGRFAMs 9.0 [69]) were used for scanning significant domains with HMMER3 [70], [71]. Blast2go [72] was used for Gene Ontology (GO) [27] and InterPro [73] annotation. Cytochrome P450 (InterPro ID: IPR001128), short-chain dehydrogenase (IPR002198), and protein kinases (for InterPro IDs see [74]) were annotated based on the result of InterPro annotation. Transcription factors were annotated according to the InterPro IDs in Fungal Transcription Factor Database [75]. Peptidases and transporters were identified and classified by BLASTp search against MEROPS 9.2 database [76] and Transport Classification Database [77], respectively, with an E-value threshold of 1E-5. Secondary metabolism gene clusters were predicted with SMURF [78]. Proteins involved in protein secretion were identified by BLASTp against *P. decumbens* protein models using the annotated proteins in *A. niger* [79] and checked manually. Orthologs of known regulators of lignocellulolytic enzymes expression were identified by bidirectional BLASTp best-hit search (E-value cutoff of 1E-10, coverage ≥ 60%) and phylogenetic analysis.

### Comparative Genomics and Phylogenetic Analysis

An all-against-all pairwise BLASTp similarity search was performed using protein models from twenty fungi (see legend of **Figure S1**). Orthologous groups were clustered using OrthoMCL [80] version 2.0 package with E-value cutoff of 1E-5 and percentage match cutoff of 50. The procedure resulted in 17,697 gene orthologous groups which contain at least two members. Multiple sequence alignments were implemented with ClustalW 2.0 [81] for 1,760 orthologous groups containing single-copy-number gene in twelve fungi (**Table S6**). 15,962 selected conserved blocks (631,726 amino acid positions from the original 1,256,803 positions) were obtained from the concatenated multiple alignments sequencing with Gblocks [82] (0.91 b) with default parameters. Phylogenetic analyses were then performed with Tree-Puzzle [83] version 5.2 for maximum likelihood analysis (25,000 puzzling steps and 4 rate categories for the discrete Gamma distribution) under the VT Model [84]. Consensus trees were graphically displayed with FigTree (http://tree.bio.ed.ac.uk/software/figtree/) version 1.3.1. For comparison of functional categories, GO of proteins in six other fungi (**Figure 1B**) were re-annotated, and the numbers of proteins in selected GO terms were visualized using customized PERL scripts. Catalytic domains of GH family 5 endo-β-1,4-glucanases in five fungi were aligned using ClustalX 2.0 [81], and then the phylogenetic tree was generated by MEGA 5.05 [85] using bootstrap maximum likelihood method with 1000 replicates.

### CAZyme Prediction, Annotation and Cluster Finding

CAZy families associated with PFAM identifiers in CAZy [31] database were assigned based on the result of PFAM domain annotation. For CAZy families without PFAM identifiers, a BLASTp search against all proteins in the CAZy database was performed with an E-value cutoff of 1E-10. A careful manual check was done in order to avoid obviously fault positive hits. CAZymes were further annotated according to results of BLASTp search against characterized proteins in CAZy database. All candidate CAZyme clusters, which begin and end with putative CAZyme-coding genes, were enumerated in the scaffolds. A total of 264 clusters were calculated as statistically significant (*P*-value ≤0.001) using the hypergeometric distribution, of which eight were highlighted in **Figure S3**.

### Protein Concentration Determination and Enzyme Assays

Protein concentration and enzyme activities were assayed as previously described [86].

### Secretome Analysis

Culture broths at 48 h were centrifuged at 12,000 × *g* at 4°C for 20 min. The supernatants were concentrated and desalted with Biomax 10,000 cutoff membrane (Millipore Corporation, USA) and 2D Clean-Up kit (GE Healthcare), and then dissolved in lysis buffer (7 M urea, 2 M thiourea, 4% wt/vol 3-((3-Cholamidopropyl)dimethylammonium)-1-propanesulfonate (CHAPS), 1% wt/vol protease inhibitor cocktail (Roche)).

DIGE analysis was performed using Ettan DIGE System (GE Healthcare) in dark. Protein samples to be compared on one gel and their equivalent mixture (as an internal standard) were labeled using different CyDye™ DIGE Fluor minimal dyes Cy3, Cy5 and Cy2 (GE Healthcare), respectively, according to the manufacturer’s instructions. In two individual experiments, the two protein samples were labeled reciprocally with Cy3 and Cy5 dyes. The three labeled protein samples are then mixed equally. 50 µg proteins were diluted to 450 µl using rehydration buffer (7 M urea, 2% w/v CHAPS, 10 mg/mL dithiothreitol (DTT) and 1% IPG buffer (pH 3–10, GE Healthcare)) and then rehydrated with Immobiline DryStrips (pH 3–10, 24 cm, GE Healthcare) in the focusing tray. Isoelectric focusing (IEF) was performed on Ettan IPGphor (GE Healthcare) system. After rehydration for 6 h at 30 V and 6 h at 60 V, IEF was carried out at 100 V for 30 min, 250 V for 1 h, 500 V for 2 h, 10,000 V for 2 h, 10,000 V for 5 h, 1,000 V for 75,000 Vh and 500 V for 24 h. After isoelectric focusing, strips were equilibrated with 10 mL equilibration buffer I (6 M urea, 50 mM Tris-HCl pH 8.8, 30% v/v glycerol, 2% w/v sodium dodecyl sulfate (SDS), 10 mg/mL DTT) for 15 min, and then equilibrated with 10 mL equilibration buffer II (the same with equilibration buffer I with 25 mg/mL iodoacetamide (Acros Organics) instead of DTT) for 10 min. The second dimensional SDS-PAGE was performed using an Ettan DALT Twelve system (GE Healthcare). Stips were loaded on 12% acrylamide gels and then sealed with low melting-point agarose. The electrophoresis ran at 10 mA per gel at 15°C for 60 min, and then at 40 mA per gel until the bromophenol blue front acheived the bottom of the gel.

Gels were scanned with Typhoon™ 9410 Variable Mode Imager (GE Healthcare) at 488/520 nm, 532/580 nm and 633/670 nm for Cy2, Cy3 and Cy5, respectively. DeCyder™ Differential Analysis Software (Version 5.02, GE Healthcare) was used to analyze images and quantify spot changes between samples. The average abundances of protein spots in two technical replicates were used for calculation of abundance ratios. For protein spots preparation, 2D electrophoresis was also performed for each sample (1 mg protein per gel) and then stained with Coomassie brilliant blue R-250. Spots of interest were manually excised, washed and trypsin (Promega)-digested for mass spectrometry. MALDI-TOF/TOF mass spectrometry was performed on 4700 Proteomics Analyzer (Applied Biosystems, USA) in the positive ion reflector mode. Peptide mass fingerprinting and peptide fragment ion data were used to search the in-house *P. decumbens* protein sequence database using the Global Proteome Server Explorer™ software (Version 3.5, Applied Biosystems).

### Accession Numbers

The Whole Genome Shotgun projects have been deposited in DDBJ/EMBL/GenBank under the accession number AGIH00000000. The raw data of 454 transcriptome sequencing have been deposited in NCBI’s Sequence Read Archive under the accession numbers SRA048521.1.

## Supporting Information

Figure S1 **Relationship of** ***P. decumbens*** **and other fungi.** (**A**) Species distribution of top hits of BLASTp search using *P. decumbens* proteins against the NCBI non-redundant protein database. (**B**) Shared and unique gene families among twenty fungal species based on orthologous groups clustering. Numbers in parentheses refer to numbers of genes in corresponding gene families. The eighteen fungi included *Saccharomyces cerevisiae*, *Schizosaccharomyces pombe*, *Aspergillus nidulans*, *Aspergillus fumigatus*, *Aspergillus niger*, *Neosartorya fischeri*, *Aspergillus oryzae*, *Aspergillus clavatus*, *Aspergillus flavus*, *Aspergillus terreus*, *Penicillium marneffei*, *Talaromyces stipitatus*, *Magnaporthe grisea*, *Chaetomium globosum*, *Fusarium graminearum*, *Neurospora crassa*, *Trichoderma reesei* and *Phanerochaete chrysosporium*.
(PDF)

Figure S2 **Comparison of number of CBM1-containing proteins in sequenced** ***Aspergillus*** **and** ***Penicillium*** **species.** “Other proteins” include putative PHB depolymerase, chitinase, etc.
(PDF)

Figure S3 **Distribution of genes encoding plant cell wall degrading-enzymes in** ***P. decumbens*** **genome.** Eight regions (clusters, C1 to C8, see **Table S11**) rich in plant cell wall degrading-enzymes are indicated.
(PDF)

Figure S4 **Extracellular enzymes production by** ***P. decumbens*** **in medium containing glucose or cellulose-wheat bran.** Different enzyme activities (**A–E**) and total protein concentration (**F**) were determined. The data represent the average of three biological replicate experiments, and error bars represent standard deviations.
(PDF)

Table S1 **Statistics of genome sequencing and assembly.**
(DOC)

Table S2 **Scaffold information of the** ***P. decumbens*** **114-2 genome.**
(DOC)

Table S3 **Statistics of gene model prediction and curation.**
(DOC)

Table S4 **Numbers of gene models with transcriptome sequencing data support and with function predictions.**
(DOC)

Table S5 **Genome statistics comparison of 12 fungal species.**
(XLS)

Table S6 **Orthologs used for phylogenetic tree construction.**
(XLS)

Table S7 **Comparison of number of proteins in level_3 Gene Ontology terms among seven fungal species.**
(XLS)

Table S8 **Comparison of numbers of selected functional proteins in** ***P. decumbens*** **,** ***P. chrysogenum*** **and** ***T. reesei*** **.**
(DOC)

Table S9 **CAZymes in** ***P. decumbens*** **.**
(XLS)

Table S10 **List of plant cell wall degrading-enzymes in five fungal species.**
(XLS)

Table S11 **List of CAZymes in genomic regions containing high density of plant cell wall degrading-enzymes in** ***P. decumbens*** **.**
(XLS)

Table S12 **Predicted secreted proteins in** ***P. decumbens*** **.**
(XLS)

Table S13 **Identification and quantification of protein spots in DIGE analysis.**
(XLS)

Table S14 **Ortholog distribution of characterized proteins involved in regulation of lignocellulolytic enzymes synthesis in addition to transcription factors in** ***P. decumbens*** **and** ***T. reesei*** **.**
(DOC)

Table S15 **Secretory pathway proteins predicted in** ***P. decumbens*** **and** ***T. reesei*** **.**
(XLS)
