# Supplementary figures and images for: Genomic and Secretomic Analyses Reveal Unique Features of the Lignocellulolytic Enzyme System of Penicillium decumbens
Source: PLoS One. 2013 Feb 1;8(2):e55185. doi: 10.1371/journal.pone.0055185 (PMC3562324; doi:10.1371/journal.pone.0055185)

A

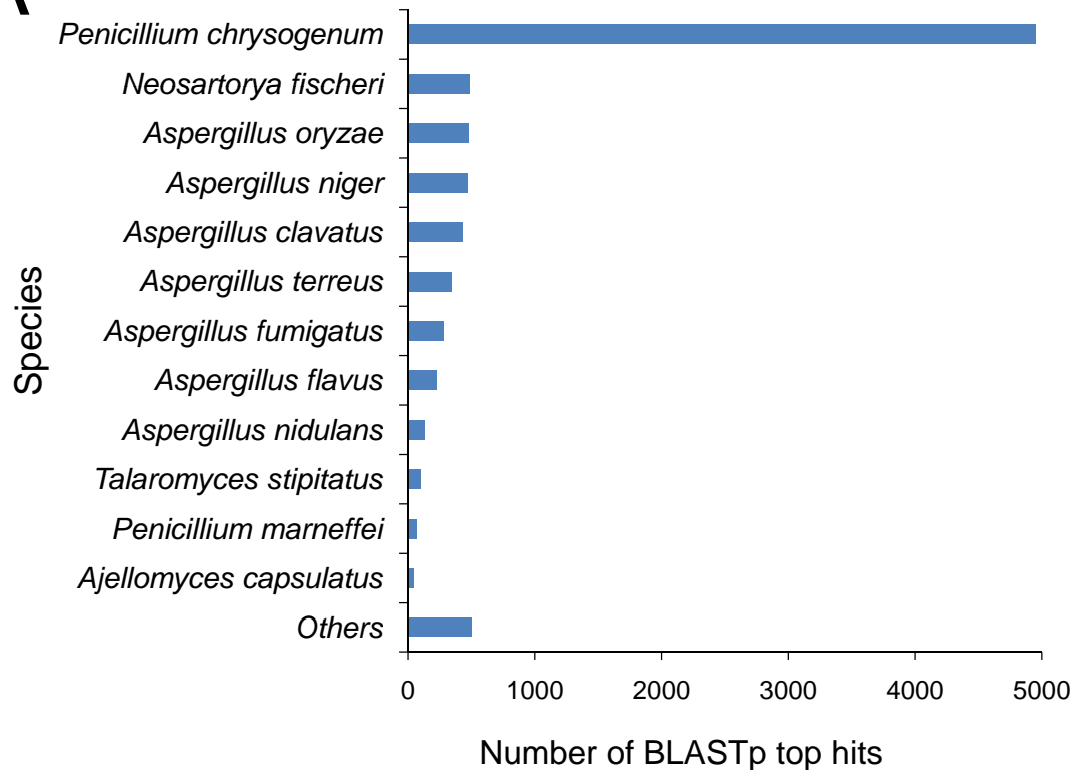

B

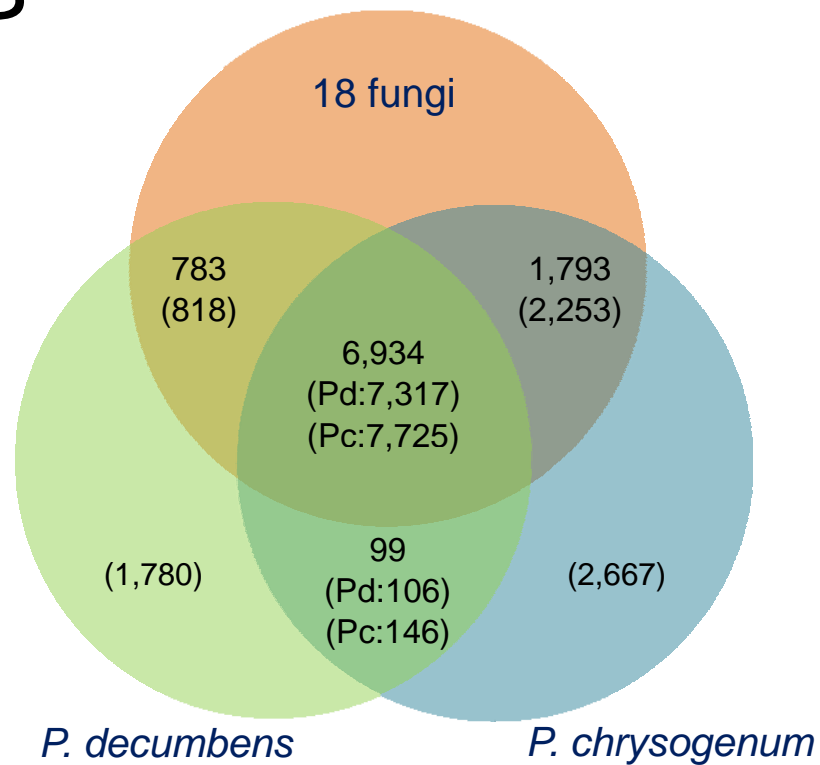

Supplement: Figure S1 — Relationship of P. decumbens and other fungi. (A) Species distribution of top hits of BLASTp search using P. decumbens proteins against the NCBI non-redundant protein database. (B) Shared and unique gene families among twenty fungal species based on orthologous groups clustering. Numbers in parentheses refer to numbers of genes in corresponding gene families. The eighteen fungi included Saccharomyces cerevisiae, Schizosaccharomyces pombe, Aspergillus nidulans, Aspergillus fumigatus, Aspergillus niger, Neosartorya fischeri, Aspergillus oryzae, Aspergillus clavatus, Aspergillus flavus, Aspergillus terreus, Penicillium marneffei, Talaromyces stipitatus, Magnaporthe grisea, Chaetomium globosum, Fusarium graminearum, Neurospora crassa, Trichoderma reesei and Phanerochaete chrysosporium. (PDF) [file pone.0055185.s001.pdf]

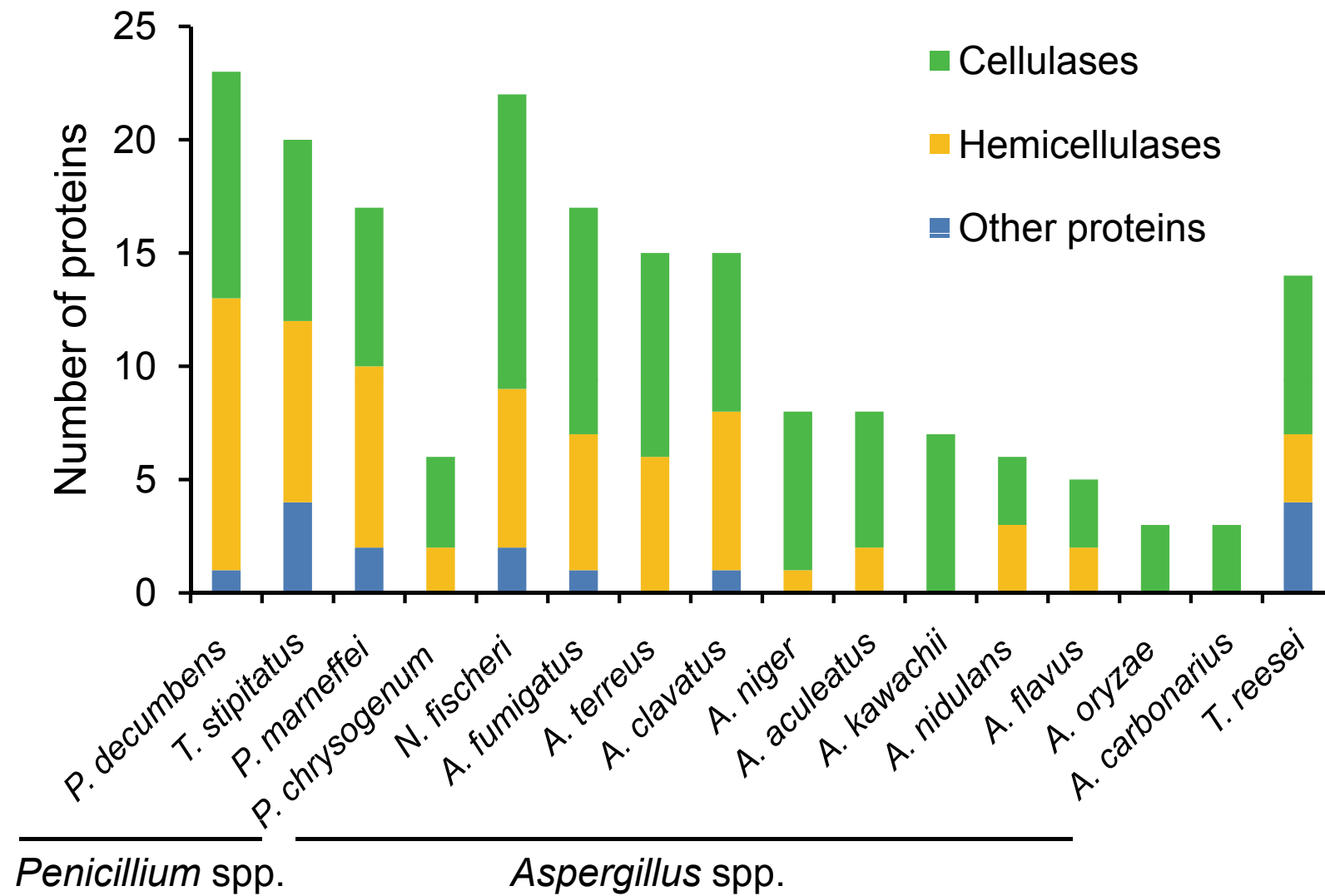

Supplement: Figure S2 — Comparison of number of CBM1-containing proteins in sequenced Aspergillus and Penicillium species. “Other proteins” include putative PHB depolymerase, chitinase, etc. (PDF) [file pone.0055185.s002.pdf]

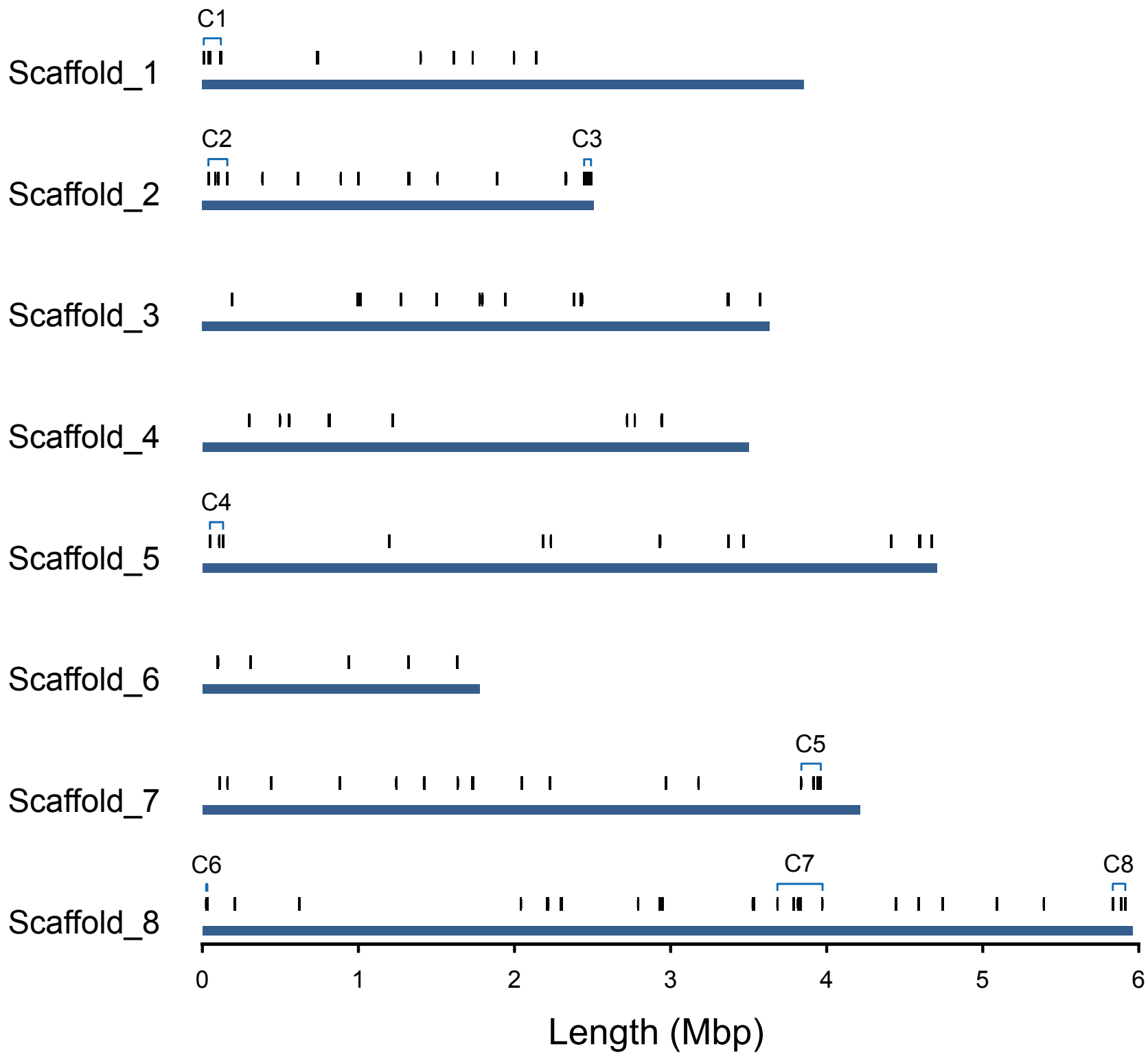

Supplement: Figure S3 — Distribution of genes encoding plant cell wall degrading-enzymes in P. decumbens genome. Eight regions (clusters, C1 to C8, see Table S11) rich in plant cell wall degrading-enzymes are indicated. (PDF) [file pone.0055185.s003.pdf]

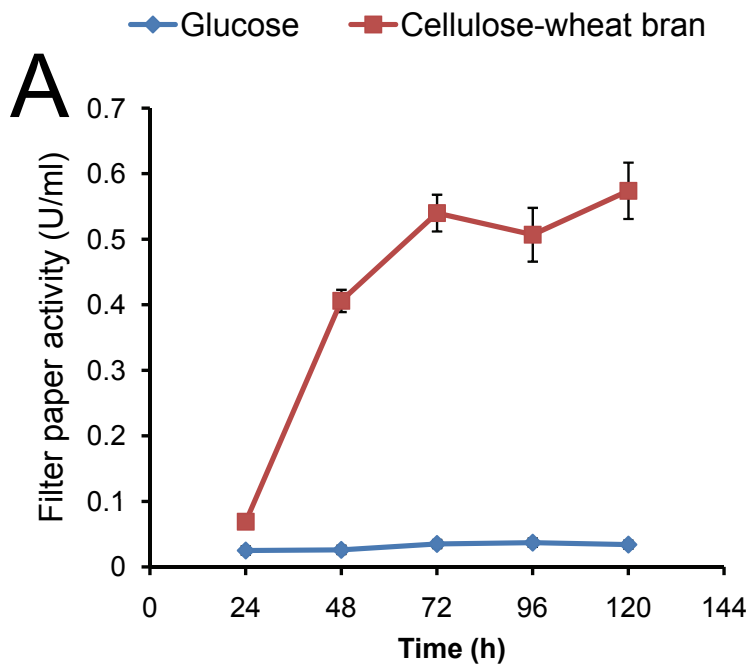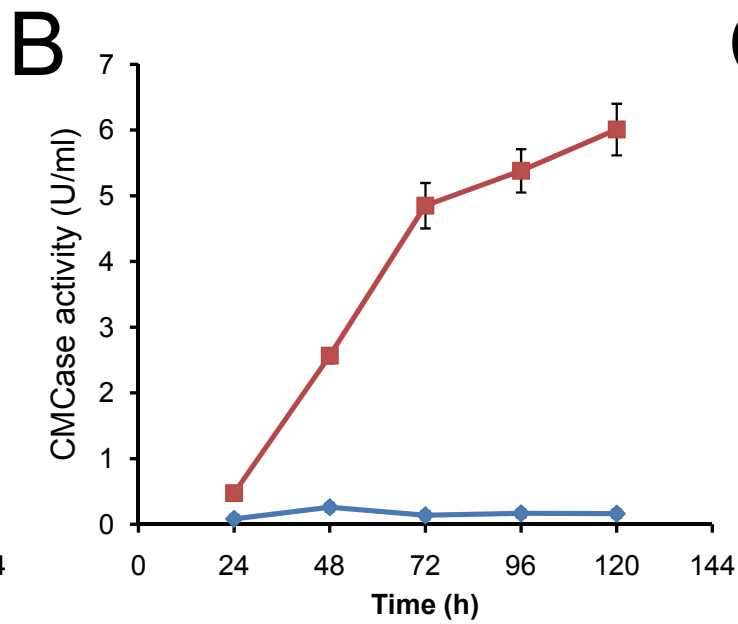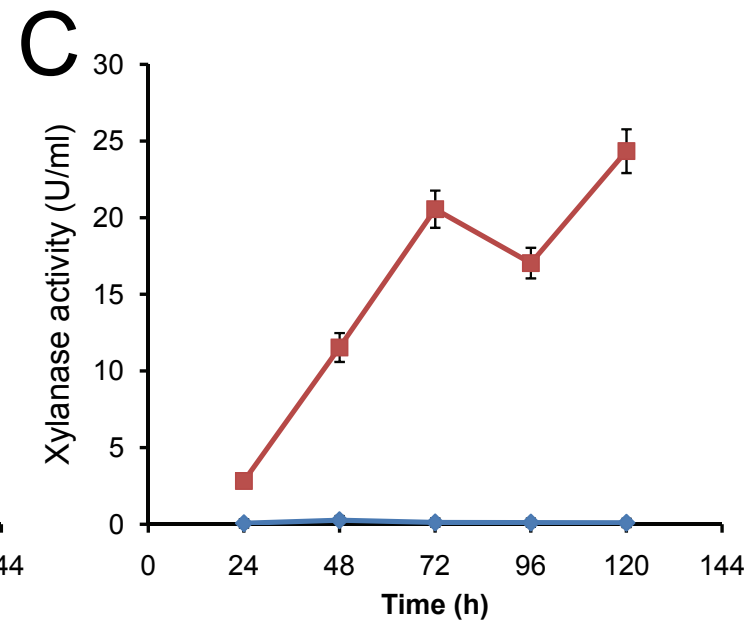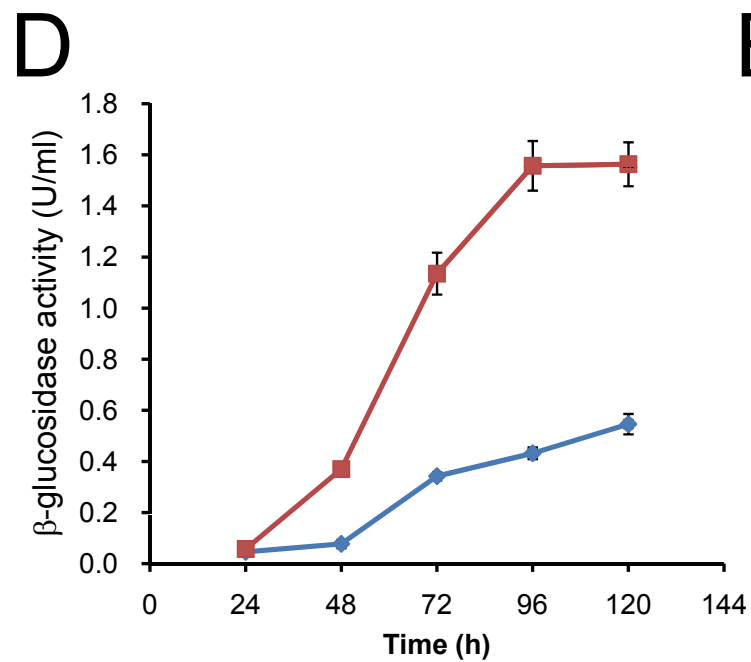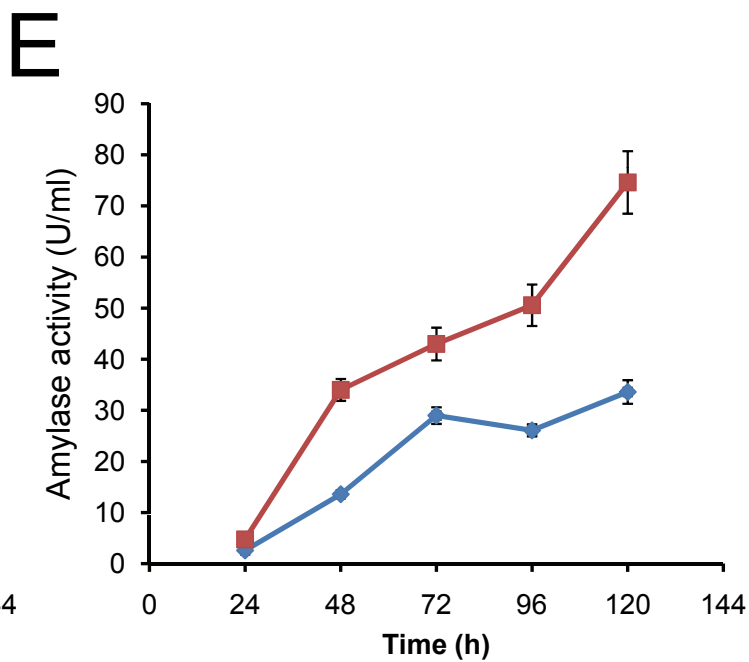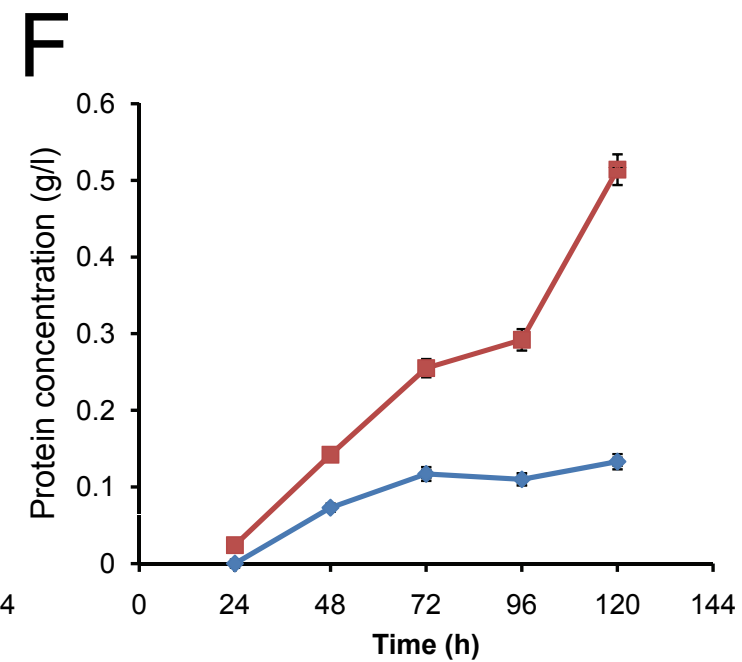

Supplement: Figure S4 — Extracellular enzymes production by P. decumbens in medium containing glucose or cellulose-wheat bran. Different enzyme activities (A–E) and total protein concentration (F) were determined. The data represent the average of three biological replicate experiments, and error bars represent standard deviations. (PDF) [file pone.0055185.s004.pdf]
