# Supplementary material for: Genomic and Secretomic Analyses Reveal Unique Features of the Lignocellulolytic Enzyme System of Penicillium decumbens
Source: PLoS One. 2013 Feb 1;8(2):e55185. doi: 10.1371/journal.pone.0055185 (PMC3562324; doi:10.1371/journal.pone.0055185)
Supplement: Table S1 — Statistics of genome sequencing and assembly. (DOC) [file pone.0055185.s005.doc]

**Table S1.** Statistics of genome sequencing and assembly.

|  | **Numbers** |
| --- | --- |
| **Sequencing** | |
| 454 single-end reads | 2,149,957 |
| 454 reads total length (bp) | 866,418,223 |
| 454 reads average length (bp) | 403 |
| SOLiD mate-paired reads | 110,118,319 |
| **Assembly** | |
| Assembly size (Mb) | 30.19 |
| Contigs | 344 |
| Scaffods | 9 |
| Contig N50 (bp) | 157,054 |
| Scaffold N50 (bp) | 3,855,354 |
