# Supplementary material for: Genomic and Secretomic Analyses Reveal Unique Features of the Lignocellulolytic Enzyme System of Penicillium decumbens
Source: PLoS One. 2013 Feb 1;8(2):e55185. doi: 10.1371/journal.pone.0055185 (PMC3562324; doi:10.1371/journal.pone.0055185)
Supplement: Table S2 — Scaffold information of the P. decumbens 114-2 genome. (DOC) [file pone.0055185.s006.doc]

**Table S2.** Scaffold information of the *P. decumbens* 114-2 genome.

| **Scaffold** | **Contig number** | **Size (bp)^a^** | **5' telomeric repeats** | **3' telomeric repeats** |
| --- | --- | --- | --- | --- |
| Scaffold_1 | 47 | 3,855,354 | YES | YES |
| Scaffold_2 | 29 | 2,508,080 | YES | YES |
| Scaffold_3 | 36 | 3,634,442 | YES | YES |
| Scaffold_4 | 43 | 3,499,614 | YES | YES |
| Scaffold_5 | 59 | 4,705,086 | YES | YES |
| Scaffold_6 | 21 | 1,775,235 | YES | NO^b^ |
| Scaffold_7 | 41 | 4,214,043 | YES | YES |
| Scaffold_8 | 65 | 5,959,152 | YES | YES |
| Scaffold_9^c^ | 4 | 26,362 | - | - |

^a^ Including gap size filled with 100 'N's.

^b^ rDNA repeats found at the sub-telomeric region.

^c^ Circular mitochondrial genome.
