# Supplementary material for: Genomic and Secretomic Analyses Reveal Unique Features of the Lignocellulolytic Enzyme System of Penicillium decumbens
Source: PLoS One. 2013 Feb 1;8(2):e55185. doi: 10.1371/journal.pone.0055185 (PMC3562324; doi:10.1371/journal.pone.0055185)
Supplement: Table S3 — Statistics of gene model prediction and curation. (DOC) [file pone.0055185.s007.doc]

**Table S3.** Statistics of gene model prediction and curation.

|  | **Completed** | **Incompleted** | **Total** | **Identical to curated models^a^** |
| --- | --- | --- | --- | --- |
| GeneId | 8,421 | 9 | 8,430 | 3,262 |
| Fgenesh | 20,208 | 5 | 20,213 | 5,010 |
| Augustus | 50,793 | 7 | 50,800 | 7,432 |
| GeneMark | 10,503 | 39 | 10,542 | 5,601 |

^a^ Totally 1,537 models were corrected with the aid of 454 transcriptome sequencing data.
