# Supplementary material for: Genomic and Secretomic Analyses Reveal Unique Features of the Lignocellulolytic Enzyme System of Penicillium decumbens
Source: PLoS One. 2013 Feb 1;8(2):e55185. doi: 10.1371/journal.pone.0055185 (PMC3562324; doi:10.1371/journal.pone.0055185)
Supplement: Table S4 — Numbers of gene models with transcriptome sequencing data support and with function predictions. (DOC) [file pone.0055185.s008.doc]

**Table S4.** Numbers of gene models with transcriptome sequencing data support and with function predictions.

|  | **Numbers** |
| --- | --- |
| **454 transcriptome sequencing data support** | |
| Coverage =100%, Identity =100% | 3,529 (35.2%) |
| Coverage ≥95%, Identity ≥95% | 4,499 (44.9%) |
| Coverage ≥70%, Identity ≥90% | 5,647 (56.4%) |
| Intron-exon boundaries supported | 13,735 (70.2%) |
| **Function annotation** | |
| NR (E-value ≤1e-5) | 8,509 |
| Swiss-Prot (E-value ≤1e-5) | 6,302 |
| KEGG (E-value ≤1e-5, Rank =30) | 3,891 |
| String (E-value ≤1e-5, Rank =5) | 5,120 |
| **Protein domain analysis** | |
| PFAM (E-value ≤1) | 7,787 |
| SUPERFAMILY (E-value ≤1e-1) | 5,828 |
| SMART (E-value ≤1e-1) | 2,628 |
| TIGRFAM (E-value ≤1e-1) | 3,214 |
