# Supplementary material for: Genomic and Secretomic Analyses Reveal Unique Features of the Lignocellulolytic Enzyme System of Penicillium decumbens
Source: PLoS One. 2013 Feb 1;8(2):e55185. doi: 10.1371/journal.pone.0055185 (PMC3562324; doi:10.1371/journal.pone.0055185)
Supplement: Table S8 — Comparison of numbers of selected functional proteins in P. decumbens , P. chrysogenum and T. reesei . (DOC) [file pone.0055185.s012.doc]

**Table S8.** Comparison of numbers of selected functional proteins in *P. decumbens*, *P. chrysogenum* and *T. reesei*.

| **Protein** | **Number of proteins** | | |
| --- | --- | --- | --- |
|  | ***P. decumbens*** | ***P. chrysogenum*** | ***T. reesei*** |
| Peptidases | 285 | 347 | 302 |
| Cytochrome P450s | 67 | 101 | 73 |
| Short-chain dehydrogenases | 85 | 129 | 126 |
| Secondary metabolism "backbone" enzymes | 39 | 49 | 27 |
| MFS transporters | 254 | 402 | 232 |
| ABC transporters | 52 | 72 | 56 |
| Protein kinases | 117 | 181 | 123 |
| Transcription factors | 476 | 584 | 484 |
