# Supplementary material for: Genomic and Secretomic Analyses Reveal Unique Features of the Lignocellulolytic Enzyme System of Penicillium decumbens
Source: PLoS One. 2013 Feb 1;8(2):e55185. doi: 10.1371/journal.pone.0055185 (PMC3562324; doi:10.1371/journal.pone.0055185)
Supplement: Table S14 — Ortholog distribution of characterized proteins involved in regulation of lignocellulolytic enzymes synthesis in addition to transcription factors in P. decumbens and T. reesei . (DOC) [file pone.0055185.s018.doc]

**Table S14.** Ortholog distribution of characterized proteins involved in regulation of lignocellulolytic enzymes synthesis in addition to transcription factors in *P. decumbens* and *T. reesei*.

| **Protein name** | **GenBank accession No.** | **Function** | **Role in lignocellulolytic enzymes expression** | **Reference(s)** | **Protein ID of orthologs** | |
| --- | --- | --- | --- | --- | --- | --- |
|  |  |  |  |  | ***P. decumbens*** | ***T. reesei*** |
| **Signaling proteins** | | | | | | |
| BLR1 (*T. reesei*) | AAV80185 | Photoreceptor white collar 1 | Transcription of cellulase gene and extracellular cellulase activity decreased in *blr1* deletion strain | [1,2] | PDE_01531 | 121962 |
| BLR2 (*T. reesei*) | AAV80186 | Photoreceptor white collar 2 | Transcription of cellulase gene decreased, but extracellular cellulase activity increased, in *blr2* deletion strain | [1,2] | PDE_08612 | 22699 |
| ENVOY (*T. reesei*) | AAT40588 | Light regulatory protein | Transcription of cellulase gene delayed in light, but extracellular cellulase activity increased, in *env1* deletion strain | [2,3] | Not found | 81609 |
| CPG-1 (*Cryphonectria parasitica*) | AAA67706 | G-protein alpha subunit | Transcription of cellulase gene deficient in *cpg-1* cosuppression strain | [4] | PDE_00146 | 123302 |
| GNA3 (*T. reesei*) | ABJ55985 | G-protein alpha subunit | Transcription of cellulase gene increased in *gna3* constitutively activation strain in light | [5] | PDE_00125 | 21505 |
| GNB1 (*T. reesei*) | EGR50145 | G-protein beta subunit | Transcription of cellulase genes decreased in *gnb1* deletion strain | [6] | PDE_07459 | 46469 |
| GNG1 (*T. reesei*) | EGR50886 | G-protein gamma subunit | Transcription of cellulase genes decreased in *gng1* deletion strain, especially in light | [6] | PDE_02978 | 75949 |
| PhLP1 (*T. reesei*) | EGR50146 | Class I phosducin-like protein interacting with G-protein beta and gamma subunit | Transcription of cellulase genes decreased in *phlp1* deletion strain, especially in light | [6] | PDE_07458 | 58856 |
| ACY1 (*T. reesei*) | EGR47772 | Adenylate cyclase | Transcription of cellulase genes decreased in *acy1* deletion strain, both in light and darkness | [7] | PDE_08988 | 124340^a^ |
| PKAC1 (*T. reesei*) | EGR50676 | cAMP dependent protein kinase A catalytic subunit 1 | Transcription of cellulase genes decreased in light, but increased in darkness, in *pkac1* deletion strain | [7] | PDE_03213 | 57399 |
| **Proteins involved in carbon catabolite repression in addition to CreA** | | | | | | |
| CreB (*A. nidulans*) | Q96V54 | Deubiquitinating enzyme | Stabilize the carbon catabolite repressor CreA | [8] | PDE_08362 | 122405 |
| CreC (*A. nidulans*) | Q9P4R5 | WD40-repeat protein | Interacts with CreB and prevents its proteolysis | [9] | PDE_08941 | 64608 |
| CreD (*A. nidulans*) | Q6SIF1 | Arrestin domains and PY motifs-containing protein | The phenotypic effects of *creB* and *creC* mutation strains were suppressed in *creD* mutation strain | [10] | PDE_08927 | 81690 |
| FbxA (*A. nidulans*) | CBF77364 | F-box containing protein genetically interacting with CreABC | Transcription of xynlanase gene and extracellular xynlanase activity decreased in *fbxA* deletion strain | [11] | PDE_05269 | 78268 |
| GlkA (*A. nidulans*) | EAA60238 | Glucokinase | Carbon catabolite derepressed in *glkA*-*hxkA* double deletion strain | [12] | PDE_03370 | 80231 |
| HxkA (*A. nidulans*) | P80581 | Hexokinase | Carbon catabolite derepressed in *glkA*-*hxkA* double deletion strain | [12] | PDE_03592 | 73665 |
| PGI (*T. reesei*) | CAG38420 | Phosphoglucose isomerase | Cellulase activity increased on glucose, but not on lactose, in *pgi1* deletion strain | [13] | PDE_03736 | 5776 |
| **Other proteins** | | | | | | |
| NCU05137 (*N. crassa*) | EAA27399 | Conserved secreted protein of unknown function | Transcription of cellulase genes increased in NCU05137 deletion strain | [14] | PDE_01641 | NO |
| NCU06650 (*N. crassa*) | EAA31647 | Secreted prokaryotic  phospholipase A2 | Cellulase activity increased in NCU06650 deletion strain | [15] | Not found | 67579 |
| GRD1 (*T. reesei*) | EGR44778 | Polyol dehydrogenase which may produce cellobiitol from cellobiose | Transcription of cellulase gene decreased in light, but not in darkness, in *grd1* deletion strain | [16] | Not found | 123946 |
| LAE1 (*T. reesei*) | AFK30952 | Protein methyltransferase | Transcription of cellulase gene and cellulase activity completely lost in *lae1* deletion strain | [17] | PDE_00584 | 41617 |

**References**

1. Castellanos F, Schmoll M, Martinez P, Tisch D, Kubicek CP, et al. (2010) Crucial factors of the light perception machinery and their impact on growth and cellulase gene transcription in *Trichoderma reesei*. Fungal Genet Biol 47: 468-476.

2. Gyalai-Korpos M, Nagy G, Mareczky Z, Schuster A, Reczey K, et al. (2010) Relevance of the light signaling machinery for cellulase expression in *Trichoderma reesei* (*Hypocrea jecorina*). BMC Res Notes 3: 330.

3. Schmoll M, Franchi L, Kubicek CP (2005) Envoy, a PAS/LOV domain protein of *Hypocrea jecorina* (Anamorph *Trichoderma reesei*), modulates cellulase gene transcription in response to light. Eukaryot Cell 4: 1998-2007.

4. Wang P, Nuss DL (1995) Induction of a *Cryphonectria parasitica* cellobiohydrolase I gene is suppressed by hypovirus infection and regulated by a GTP-binding-protein-linked signaling pathway involved in fungal pathogenesis. Proc Natl Acad Sci U S A 92: 11529-11533.

5. Schmoll M, Schuster A, Silva Rdo N, Kubicek CP (2009) The G-alpha protein GNA3 of *Hypocrea jecorina* (Anamorph *Trichoderma reesei*) regulates cellulase gene expression in the presence of light. Eukaryot Cell 8: 410-420.

6. Tisch D, Kubicek CP, Schmoll M (2011) The phosducin-like protein PhLP1 impacts regulation of glycoside hydrolases and light response in *Trichoderma reesei.* BMC Genomics 12: 613.

7. Schuster A, Tisch D, Seidl-Seiboth V, Kubicek CP, Schmoll M (2012) The role of protein kinase A and adenylate cyclase in light-modulated cellulase regulation in *Trichoderma reesei*. Appl Environ Microbiol.

8. Lockington RA, Kelly JM (2001) Carbon catabolite repression in *Aspergillus nidulans* involves deubiquitination. Mol Microbiol 40: 1311-1321.

9. Lockington RA, Kelly JM (2002) The WD40-repeat protein CreC interacts with and stabilizes the deubiquitinating enzyme CreB in vivo in *Aspergillus nidulans*. Mol Microbiol 43: 1173-1182.

10. Boase NA, Kelly JM (2004) A role for *creD*, a carbon catabolite repression gene from *Aspergillus nidulans*, in ubiquitination. Mol Microbiol 53: 929-940.

11. Colabardini AC, Humanes AC, Gouvea PF, Savoldi M, Goldman MH, et al. (2011) Molecular characterization of the *Aspergillus nidulans fbxA* encoding an F-box protein involved in xylanase induction. Fungal Genet Biol 49: 130-140.

12. Flipphi M, van de Vondervoort PJ, Ruijter GJ, Visser J, Arst HN, Jr., et al. (2003) Onset of carbon catabolite repression in *Aspergillus nidulans*. Parallel involvement of hexokinase and glucokinase in sugar signaling. J Biol Chem 278: 11849-11857.

13. Limon MC, Pakula T, Saloheimo M, Penttila M (2011) The effects of disruption of phosphoglucose isomerase gene on carbon utilisation and cellulase production in *Trichoderma reesei* Rut-C30. Microb Cell Fact 10: 40.

14. Tian C, Beeson WT, Iavarone AT, Sun J, Marletta MA, et al. (2009) Systems analysis of plant cell wall degradation by the model filamentous fungus *Neurospora crassa*. Proc Natl Acad Sci U S A 106: 22157-22162.

15. Sun J, Glass NL (2011) Identification of the CRE-1 cellulolytic regulon in *Neurospora crassa*. PLoS One 6: e25654.

16. Schuster A, Kubicek CP, Schmoll M (2011) Dehydrogenase GRD1 represents a novel component of the cellulase regulon in *Trichoderma reesei* (*Hypocrea jecorina*). Appl Environ Microbiol 77: 4553-4563.

17. Seiboth B, Karimi RA, Phatale PA, Linke R, Hartl L, et al. (2012) The putative protein methyltransferase LAE1 controls cellulase gene expression in *Trichoderma reesei*. Mol Microbiol 84: 1150-1164.
